# Changes in hemorrhage pattern on consecutive non-contrast CT scans in non-aneurysmal subarachnoid hemorrhage patients

**DOI:** 10.1016/j.bas.2025.105603

**Published:** 2025-09-06

**Authors:** René van den Berg, Wouter Dronkers, Olvert Berkhemer, Arian Karbe, Menno Germans, W. Peter Vandertop, Bart J. Emmer, Dagmar Verbaan

**Affiliations:** a Amsterdam University Medical Centers, Location AMC, Department of Radiology and Nuclear Medicine, Amsterdam, the Netherlands; b Amsterdam University Medical Centers, Location AMC, Department of Neurosurgery, Amsterdam, the Netherlands; c University Hospital Zürich, University of Zürich, Department of Neurosurgery, Zürich, Switzerland; d Amsterdam Neuroscience, Neurovascular Disorders, Amsterdam, the Netherlands

**Keywords:** Subarachnoid hemorrhage, Perimesencephalic hemorrhage, Non contrast CT

## Abstract

**Introduction:**

The diagnosis perimesencephalic subarachnoid hemorrhage (PMSAH) is based on a specific distribution pattern within 72 h after ictus. However solid evidence is lacking for this time window with the potential risk that blood distribution can change over time, with implications for the management of these patients.

**Research question:**

To study cisternal and intraventricular blood pattern changes on initial and follow-up non-contrast CT scans (NCCTs) during the first 72 h.

**Materials and methods:**

This retrospective study included consecutive PMSAH and non-perimesencephalic (NPSAH) patients in whom at least two NCCTs were performed within the first 72 h. Presence and changes in the distribution of cisternal and intraventricular blood was independently assessed by three observers.

**Results:**

135 patients (62 PMSAH and 73 NPSAH) were included. The distribution of SAH remained unchanged within the first 72-h in 63 (47 %) patients (PMSAH: 38 (61 %), NPSAH: 25 (39 %))(p < 0.01). An increase in SAH distribution from 0 to 6 h was seen in 7/47 PMSAH (15 %) versus 24/47 (51 %) in NPSAH patients (p < 0.01). A decrease in SAH distribution from 0 to 6 h was seen in 6/47 (13 %) PMSAH patients versus 2/47 (4 %) NPSAH patients (p < 0.01). Between 6 and 24 h, a 72 % decrease (10/14) was only seen in NPSAH patients. The diagnosis PMSAH never changed to NPSAH or vice versa due to SAH redistribution.

**Discussion and conclusion:**

Within the first 72 h after ictus, redistribution of cisternal and intraventricular blood is seen more often in NPSAH than in PMSAH patients but did not change the specific diagnosis.

## Highlights

• Spreading of SAH can alter the diagnosis from PMSAH into NPSAH.
• The altered diagnosis also changes the management.
• SAH distribution in PMSAH patients is more stable than in NPSAH patients.
• The diagnosis NPSAH or PMSAH remains unchanged during the first 72 h.

## 1 Introduction

Non-aneurysmal subarachnoid hemorrhage (NASAH) can be divided into perimesencephalic- (PMSAH) and non-perimesencephalic subarachnoid hemorrhage (NPSAH). The diagnosis PMSAH in which there is a characteristic pattern of blood on non-contrast computed tomography (NCCT) in the absence of an aneurysm on vascular imaging was established by Rinkel et al. (van Gijn and van Dongen, 1982; Rinkel et al., 1991a; Rinkel et al., 1991b). At the turn of the century an invasive digital subtraction angiography (DSA) was still advocated to exclude the presence of an aneurysm because the risk of a fatal rebleed from a non-diagnosed aneurysm clearly outweighed the complications of DSA (Velthuis et al., 1999). However, with the advent of CT-angiography (CTA) and subsequent improvement of the diagnostic accuracy after introduction of multidetector CT-scanners, the diagnosis PMSAH can also be made without invasive imaging (Velthuis et al., 1999; Agid et al., 2010; Kelliny et al., 2011). Besides the different approaches on the value of CTA versus DSA in patients with a perimesencephalic blood distribution (Hoh et al., 2023; Steiner et al., 2013) the change in distribution of cisternal and ventricular blood within the first 72 h has never been addressed and radiological evidence supporting this 72 h cut-off point is lacking (Rinkel et al., 1991b; Kapadia et al., 2014; Mensing et al., 2018). Increase in distribution of blood towards the lateral Sylvian and interhemispheric fissures within the first 72 h could alter the diagnosis from PMSAH into NPSAH, with the consequence that a DSA is needed to exclude an underlying vascular lesion (Hoh et al., 2023). Vice versa, rapid resorption of subarachnoid blood could lead to underreporting of NPSAH and culprit vascular lesions might be missed. In addition, the risk to develop SAH-related complications, such as delayed cerebral ischemia and hydrocephalus, is also different for PMSAH and NPSAH (Mensing et al., 2018; Konczalla et al., 2015).

We therefore reviewed all NCCT scans in NASAH patients for changes in the SAH distribution pattern from the first admission NCCT scan compared to follow-up scans within the first 72 h after ictus to determine if these specific diagnoses remain unchanged.

## 2 Methods

### 2.1 Patient population

From all consecutive patients in our SAH registry, we reviewed 135 NASAH patients with at least two NCCT scans within the first 72 h after diagnosis, in whom no aneurysmal or other cause of SAH was found. Patients were included in the period between January 1st^,^ 2012 up to December 31st^,^ 2020 to Centre 1 (n = 90) and between January 1st^,^ 2009 up to December 31st^,^ 2020 to Centre 2 (n = 63). Both institutions are large tertiary referral centers for SAH patients. Inclusion criteria were: 1) a proven SAH on the first (admission) NCCT scan performed within 24 h after ictus; 2) at least one follow-up NCCT scan performed within the first 72 h after ictus; 3) exclusion of an aneurysmal or other vascular cause by either CT-angiography or DSA. In patients with a typical PMSAH a CT-angiography was performed to exclude an aneurysmal cause of bleeding. All other included patients were stratified as NPSAH and underwent a CT-angiography, DSA and repeat DSA.

Patients were excluded if: 1) the time from ictus to the first NCCT was beyond 24 h or if time of ictus was unknown; 2) distribution of the SAH could not be determined because of poor NCCT quality; or 3) when a ventricular or lumbar drain was placed within the first 72 h as that might have influenced the volume and distribution of blood.

We intentionally did not include patients with an aneurysmal SAH because a rebleed would have disrupted findings.

### 2.2 Data collection

The following patient data were retrieved: sex, age at time of admission, date and time of ictus, first and repeat NCCT scan. All data was collected retrospectively with an emphasis on NCCT findings. Other interventions such as placement of a ventricular drain or lumbar puncture during the clinical course were assessed for correct patient inclusion. SAH-related complications were recorded, including rebleeding and development of hydrocephalus.

### 2.3 Radiological assessment

To determine the distribution pattern of SAH (PMSAH versus NPSAH), NCCT scans were scored by three independent readers, In case of discrepancies between observers, consensus reading was performed. PMSAH was defined according to the established distribution pattern (Rinkel et al., 1991b, 1993): anterior to the mesencephalon or pons, with possible extension to the perimesencephalic, ambient and suprasellar (chiasmatic) cisterns, cerebello-pontine angle, foramen magnum, proximal part of the anterior interhemispheric fissure and basal part of the Sylvian fissure. When SAH extended beyond these compartments (e.g. lateral Sylvian fissure), it was diagnosed as NPSAH. The presence of intraventricular blood was further subdivided into extensive intraventricular blood (as seen in an aneurysmal SAH) or slight sedimentation of blood (in concordance with the PMSAH definition). All these specific SAH locations were specified in a scoring form (see supplemental file). Per patient, the presence or absence of blood for each separate location was assessed for each NCCT at the different time moments. Differences between the initial and repeat scan were determined qualitatively per location; equal, increase or decrease in the distribution of cisternal or intraventricular blood. When more than one repeat NCCT scan was performed during follow-up within the 72 h time window, the latter repeat scan was used to determine changes in the distribution of cisternal or intraventricular blood.

### 2.4 Statistical analysis

All data was transferred to a database (IBM SPSS Statistics Version 28.0.1.1). Continuous variables were checked for normality using the Shapiro-Wilk test (>0.9 is considered a normal distribution). Normally distributed continuous variables were reported as means ± range, not normally distributed variables were reported as median (interquartile range (IQR)), and categorical variables as frequencies with percentages. For comparison between groups, a two sample *t*-test was used. Cross table analysis was performed with calculation of significance using the Chi-Square test.

## 3 Results

In 135 patients (62 PMSAH and 73 NPSAH) two or more NCCTs were available with a second scan performed within 72-h. The median age was 56 years (IQR 49–65) (PMSAH 55 (IQR 46–61); NPSAH 59 (range 51–68) and 55 (41 %) were female (PMSAH 30 (48 %), NPSAH (25 (34 %)).

The median time interval between the first and second scan was 3 h (IQR 1–9). One hundred-eleven (82 %) patients had a follow-up scan within 0–24 h and 24 (18 %) within 24–72 h.

Changes in the distribution of subarachnoid blood per time interval for PMSAH and NPSAH patients are presented in Table 1. During the entire interval period of 72 h, SAH distribution was unchanged in 63 (47 %) patients (38 PMSAH (61 %), 25 NPSAH (39 %))(p < 0.01). Within the first 6 h an increase in the distribution of cisternal blood was seen in 7/47 (15 %) PMSAH patients versus 24/47 (51 %) in NPSAH patients (p < 0.01. A more rapid decrease in distribution (‘disappearance’) of cisternal blood was seen within the first 6 h in 6/47 (13 %) PMSAH patients versus only in 2/47 (4 %) NPSAH patients (p < 0.01). In the subsequent 6–24 h period a 72 % decrease (10/14) was only seen in NPSAH patients. From 24 to 72 h, the change in distribution of SAH was similar for PMSAH and NPSAH patients. More detailed information on the changes in distribution per cisternal location are given in Supplemental Table 1.

**Table 1:** Changes in the distribution of subarachnoid blood per time interval for PMSAH and NPSAH patients.

Diagnosis	Interval period	Change in SAH Distribution			Total
		Equal	Increase	Decrease	
PMSAH	0–6 h	34 (72 %)	7 (15 %)	6 (13 %)	47
	6–24 h	3 (100 %)	0	0	3
	24–72 h	2 (17 %)	2 (17 %)	8 (66 %)	12
	Total	39	9	14	62
NPSAH	0–6 h	21 (45 %)	24 (51 %)	2 (4 %)	47
	6–24 h	2 (14 %)	2 (14 %)	10 (72 %)	14
	24–72 h	2 (17 %)	2 (17 %)	8 (66 %)	12
	Total	25	28	20	73

Note: SAH = subarachnoid hemorrhage, PMSAH = perimesencephalic SAH, NPSAH = non perimesencephalic SAH.

Despite the change in distribution of SAH during the entire time interval, the diagnosis PMSAH did not change to NPSAH, and vice versa, in any of the patients in this study.

**Fig. 1:**
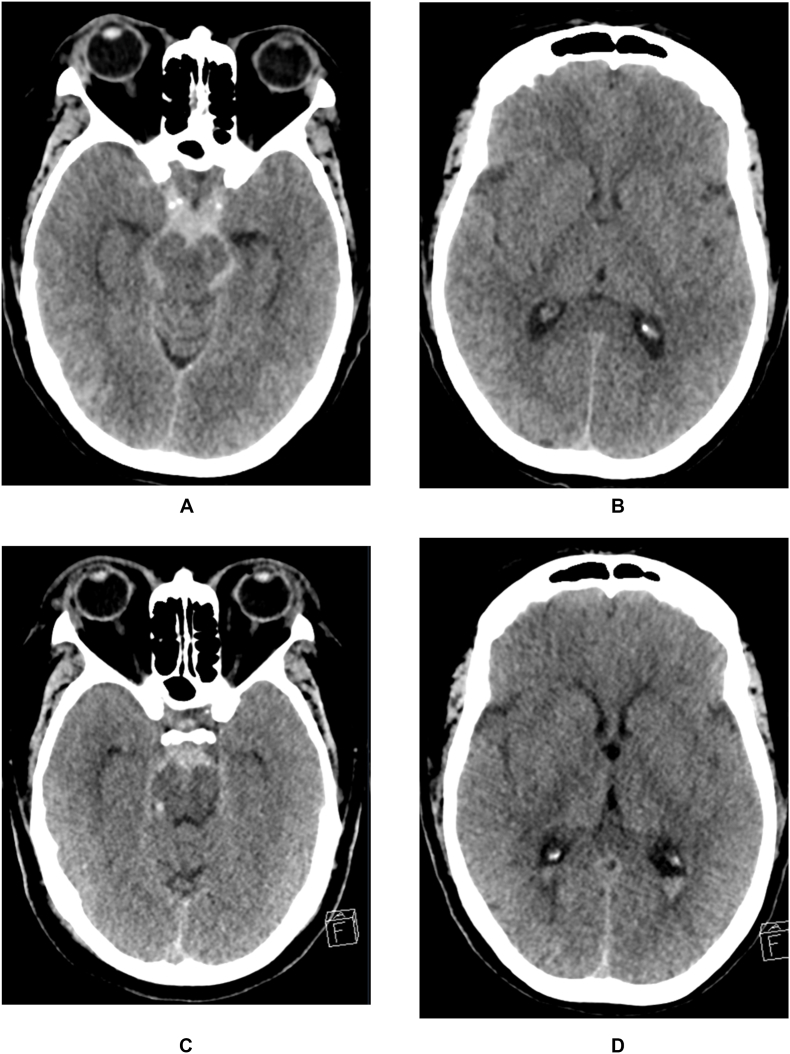
56-year-old female patient with non-aneurysmal perimesencephalic subarachnoid hemorrhage (PMSAH). On the initial non-contrast CT (NCCT) scan a significant amount of blood is seen in the suprasellar and ambient cistern (A), but absent intraventricular blood (B). On the repeat NCCT 34 h later, the amount of blood in the suprasellar cistern has decreased (C) with now presence of intraventricular blood (D).

The presence and distribution over time of intraventricular blood is presented in Table 2. On the initial or follow-up NCCT scan, intraventricular blood was seen in 16/62 PMSAH patients (26 %) and in 50/73 NPSAH patients (68 %) (p < 0.01). When the initial NCCT scan did not show intraventricular blood, de novo occurrence of intraventricular blood was seen in 6/52 (11 %) of PMSAH patients compared to 10/33 (30 %) of NPSAH patients (p < 0.01). ‘De novo intraventricular blood’ in the six PMSAH patients was only sparse sedimentation of blood (Fig. 1).

**Table 2:** Changes in the distribution of intraventricular blood for PMSAH and NPSAH patients.

Diagnosis	Initial IV Hx	Change intraventricular Hemorrhage			
		Equal	Increase	Decrease	Total
PMSAH	No	46 (89 %)	6^a^ (11 %)	NA	52 (85 %)
	Yes	4 (44 %)	4 (44 %)	2 (11 %)	10 (15 %)
	Total	50	10	2	62
NPSAH	No	23 (70 %)	10 (30 %)	NA	33 (45 %)
	Yes	32 (78 %)	7 (17 %)	1 (5 %)	40 (55 %)
	Total	55	17	1	73

Note: IV Hx = intraventricular hemorrhage, PMSAH = perimesencephalic SAH, NPSAH = non perimesencephalic SAH, NA = not applicable.

^a^ Only small sediment of blood in 4th or lateral ventricles (within definition of PMSAH).

## 4 Discussion

During the first 72 h after ictus the distribution of SAH remains stationary in roughly half of the patients but more so in PMSAH than in NPSAH patients. More importantly, when redistribution or resorption of cisternal or intraventricular blood occurs, the diagnosis did not change from PMSAH into NPSAH, or vice versa. Therefore, the 72 h time window, which has been used as a cut-off point in patients with a perimesencephalic subarachnoid hemorrhage (PMSAH), is sound and can be ratified based on the findings in this retrospective study.

The first report on the peculiar pattern of subarachnoid hemorrhage in patients without an underlying intracranial aneurysm hemorrhage goes back almost four decades and the term perimesencephalic hemorrhage (PMSAH) was introduced (van Gijn et al., 1985). In this study, patients were described without aneurysms with distinct basal hemorrhages mainly located in the perimesencephalic cisterns. The distribution pattern of SAH was later refined to the definition that is now used in guidelines (Rinkel et al., 1991b; Hoh et al., 2023; Steiner et al., 2013).

Over time, the distribution pattern of SAH in the cisterns can change and it was estimated that the probability of recognizing an aneurysmal hemorrhage on NCCT is 85 % after five days and 50 % after one week (van Gijn and van Dongen, 1982). Findings from our study show that already during the first 72 h after ictus the distribution of SAH in the cisternal spaces can change. An increase in spreading of blood is more often seen in NPSAH patients in the first 24 h after ictus. A decrease in SAH distribution in this study has been noticed in the first 6 h interval period, this can be explained by the usually much smaller amount of (often prepontine) SAH blood in these patients. A decrease in the spreading of blood is seen between 24 and 72 h for both NPSAH and PMSAH patients. Unfortunately, it is not possible to assign these changes completely to PMSAH or NPSAH alone because there is still overlap. Routine control NCCT is therefore not of value to increase the diagnostic certainty to differentiate PMSAH from NPSAH.

The key diagnostic difference between PMSAH and NPSAH on a NCCT scan is the extension of SAH into the lateral and interhemispheric fissures and beyond, or frank bleeding in the ventricles. Although in both patient groups a causative vascular lesion is not diagnosed with vascular imaging, different causes have been suggested for PMSAH and NPSAH. For PMSAH, a venous origin of the bleeding has been suggested. Contrary, for NPSAH a higher likelihood of arterial causes have been suggested, including tiny basilar artery perforator aneurysms (Granja et al., 2020). These aneurysms are difficult to diagnose, easily missed even with DSA, and additionally show a high rate of spontaneous resolution. Other causes of SAH are vascular or neoplastic lesions of the brain and spinal cord. In addition, antiplatelet or anticoagulation therapy, hypertension, and the use of sympathomimetic drugs may be held responsible for NASAH (Howard et al., 2019).

The more peripheral distribution of NPSAH versus PMSAH might be related to a higher (arterial) impact, which might force SAH from the more basal cisternal compartments to the more peripheral insular and interhemispheric cisterns. Once cisternal boundaries are traversed, an easier secondary distribution over the convexity might occur. The compartmentalization of the cisterns was already observed by Yasargil et al., with thicker and tougher arachnoid fibers and membranes where arteries pass through the trabeculated wall from one compartment to the other (Yasargil et al., 1976). The cisternal compartmentalization might explain the difference in behavior of subarachnoid blood between PMSAH and NPSAH; during the first 24 h after ictus subarachnoid blood in the Sylvian fissure and interhemispheric fissure only tends to spread over the convexity in NPSAH patients. In PMSAH patients the subarachnoid blood remains in the basal cisterns, which includes also the Sylvian cistern (Yasargil et al., 1976).

In our study population a change in intraventricular distribution was observed in less than a quarter of all patients, with either a small amount of ‘de novo’ intraventricular blood, or by extension of a small amount of intraventricular blood from the 4th to the lateral ventricles. According to the original definition of a PMSAH, such small amounts of intraventricular blood are well accepted and should not alter the diagnosis from PMSAH into NPSAH (Rinkel et al., 1991b). Only when a significant (‘frank’) amount of blood is seen initially or later on during the early stages after ictus will this change the diagnosis from PMSAH to NPSAH. In our population, an increase of intraventricular blood was observed in a small minority of patients, but in all cases this was just minor sedimentation (Fig. 1). When reporting on the presence of intraventricular blood, the amount of blood should not be overstated because it might lead to an over-diagnosis of NPSAH, with the necessity to perform a DSA to rule out a vascular cause of the SAH (Mensing et al., 2018).

The major problem in defining PMSAH versus NPSAH lies in defining the exact anatomical transition zone from the basal part towards the insular part of the Sylvian fissure and not so much in a time-related redistribution of SAH. A recent article on interrater and intra-rater reliability showed a lack in consistency to make the diagnosis PMSAH versus NPSAH (Benomar et al., 2024). In this article a ‘grey zone area’ is introduced, in which the diagnosis PMSAH or NPSAH is difficult to make, because blood extends to the borders of where we still accept blood to be present in PMSAH patients. The importance of making the right diagnosis is that the clinical course in PMSAH-patients is much more benign than in patients with NPSAH (Konczalla et al., 2015; Al-Mufti et al., 2018; Atchie et al., 2019).

The major limitation of the study, besides its retrospective nature, is the short time interval between the first and follow-up NCCT scan. Despite the fact that we were able to include all consecutive NASAH patients over an 11 year time period, three-quarters of the follow-up scans were performed within the first 9 h after ictus. These early repeat NCCT scans were performed after referral to our tertiary center in the diagnostic workup that also included a CT angiography of the head. Moreover, only few patients with a PMSAH have an indication for a repeat NCCT scan because the clinical course is much more benign. Nevertheless, we were able to identify pattern changes, both in the cisterns as well as in the ventricles. Because patients in whom a ventricular drain was placed were excluded, we might have introduced a bias and missed patients with a more severe increase in the spreading of SAH. By excluding patients with an aneurysmal or other vascular cause of the SAH we hoped to limit the possibility that a rebleed would be the cause of an increase in the distribution of SAH. Finally, we did not look into the clinical records of the patients with the very slight possibility that we missed a clinical rebleed as cause of deterioration, but the policy in our hospital is that in case of clinical deterioration after SAH, we perform a control NCCT, so the consequence to introduce a bias in this study is only minor. As the goal of this study was to focus on radiological NCCT findings during follow-up, clinical outcome differences between the two groups were not assessed.

In conclusion, this study shows that the distribution pattern of SAH in PMSAH patients is much more stable during the first 24 h after ictus than in NPSAH patients. Although the SAH distribution pattern changes in NASAH patients the diagnosis NPSAH to PMSAH remains unchanged during the first 72 h.

## Informed consent:

Informed consent was not sought for the present study because this was a blinded retrospective observational study which did not affect the management in the studied patients.

## Ethical approval

For this observational study, the Ethical Committee of the Amsterdam University Medical Center waived the necessity for formal approval because this was a retrospective blinded study.

Trial registration: not applicable because this is not a randomized trial.

## Funding

This research received no specific grant from any funding agency in the public, commercial, or not-for-profit sectors.

## Conflicting interests

The Author(s) declare(s) that there is no conflict of interest.
