# Supplementary material for: Changes in hemorrhage pattern on consecutive non-contrast CT scans in non-aneurysmal subarachnoid hemorrhage patients
Source: Brain Spine. 2025 Sep 6;5:105603. doi: 10.1016/j.bas.2025.105603 (PMC12466301; doi:10.1016/j.bas.2025.105603)
Supplement: Multimedia component 1 [file mmc1.docx]

Supplemental Table 1
Changes in distribution of subarachnoid blood per cisternal location for perimesencephalic
(PMSAH) and non perimesencephalic (NPSAH) hemorrhage.

|  | | PMSAH | NPSAH | |
| --- | --- | --- | --- | --- |
| Prepontine | Increase of blood | 1 | 0 | |
|  | Decrease of blood | 9 | 12 | |
|  | Equal blood | 52 | 61 | |
| Cerebellopontine  Right | Decrease of blood | 5 | 5 | |
|  | Equal blood | 57 | 68 | |
| Cerebellopontine  Left | Increase of blood | 2 | 0 | |
|  | Decrease of blood | 3 | 7 | |
|  | Equal blood | 57 | 66 | |
| Perimesencephalic | Increase of blood | 2 | 0 | |
|  | Decrease of blood | 9 | 12 | |
|  | Equal blood | 51 | 61 | |
| Pentagon | Increase of blood | 1 | 0 | |
|  | Decrease of blood | 10 | 9 | |
|  | Equal blood | 51 | 64 | |
| M1 segment Right | Increase of blood | 2 | 1 | |
|  | Decrease of blood | 6 | 12 | |
|  | Equal blood | 54 | 60 | |
| M1 segment Left | Increase of blood | 3 | 2 | |
|  | Decrease of blood | 6 | 10 | |
|  | Equal blood | 53 | 61 | |
| Fissure Right | Increase of blood | 1 | 2 | |
|  | Decrease of blood | 1 | 8 | |
|  | Equal blood | 60 | 63 | |
| Fissure Left | Increase of blood | 0 | 3 | |
|  | Decrease of blood | 0 | 8 | |
|  | Equal blood | 62 | 62 | |
| Hemispheric Right | Increase of blood | 2 | 5 | |
|  | Decrease of blood | 0 | 1 | |
|  | Equal blood | 60 | 67 | |
| Hemispheric Left | Increase of blood | 1 | 3 | |
|  | Decrease of blood | 0 | 2 | |
|  | Equal blood | 61 | 68 | |
| Inter-hemispheric | Increase of blood | 0 | 4 |  |
|  | Decrease of blood | 0 | 4 |  |
|  | Equal blood | 62 | 65 |  |
| Pericallosal | Increase of blood | 0 | 1 | |
|  | Decrease of blood | 0 | 1 | |
|  | Equal blood | 62 | 71 | |
