# Supplementary material for: Changes in hemorrhage pattern on consecutive non-contrast CT scans in non-aneurysmal subarachnoid hemorrhage patients
Source: Brain Spine. 2025 Sep 6;5:105603. doi: 10.1016/j.bas.2025.105603 (PMC12466301; doi:10.1016/j.bas.2025.105603)
Supplement: Multimedia component 2 [file mmc2.docx]

Supplemental file 1

Presence of subarachnoid blood per location and change over time (scoring form)

| **Patient Identification Number** | Date scan 1 | Date scan 2 |  |
| --- | --- | --- | --- |
|  |  |  |  |
|  | **Subarachnoid blood** | **Subarachnoid blood** | **Difference** |
|  | **Presence = 1** | **Presence = 1** | **Increase = 0** |
|  | **Absence = 0** | **Absence = 0** | **Decrease = 1** |
|  |  |  | **Equal = 2** |
| **Posterior Fossa** |  |  |  |
| Prepontine |  |  |  |
| Cerebello pontine Right |  |  |  |
| Cerebello pontine Left |  |  |  |
| Perimesencephalic |  |  |  |
| **Supratentorial** |  |  |  |
| Suprasellar |  |  |  |
| Basal part Sylvian fissure Right |  |  |  |
| Basal part Sylvian fissure Left |  |  |  |
| Lateral part Sylvian fissure Right |  |  |  |
| Lateral part Sylvian fissure Left |  |  |  |
| Hemispheric Right |  |  |  |
| Hemispheric Left |  |  |  |
| Interhemispheric |  |  |  |
| Pericallosal |  |  |  |
| **Ventricle** |  |  |  |
| 4th Ventricle |  |  |  |
| 3rd Ventricle |  |  |  |
| Lateral Ventricle Right |  |  |  |
| Lateral Ventricle Left |  |  |  |
| **Lobar hematoma** |  |  |  |
| **Subdural hematoma** |  |  |  |
